# Identification of a Small Molecule Inhibitor of RAD52 by Structure-Based Selection

**DOI:** 10.1371/journal.pone.0147230

**Published:** 2016-01-19

**Authors:** Katherine Sullivan, Kimberly Cramer-Morales, Daniel L. McElroy, David A. Ostrov, Kimberly Haas, Wayne Childers, Robert Hromas, Tomasz Skorski

**Affiliations:** 1 Department of Microbiology and Immunology and Fels Institute for Cancer Research and Molecular Biology, Temple University School of Medicine, Philadelphia, Pennsylvania 19140, United States of America; 2 Department of Pathology, Immunology and Laboratory Medicine, College of Medicine, University of Florida & Shands, Gainesville, Florida 32610, United States of America; 3 Moulder Center for Drug Discovery Research, Temple University School of Pharmacy, Philadelphia, Pennsylvania 19140, United States of America; 4 Department of Medicine, College of Medicine, University of Florida & Shands, Gainesville, Florida 32610, United States of America; Saint Louis University, UNITED STATES

## Abstract

It has been reported that inhibition of RAD52 either by specific shRNA or a small peptide aptamer induced synthetic lethality in tumor cell lines carrying BRCA1 and BRCA2 inactivating mutations. Molecular docking was used to screen two chemical libraries: 1) 1,217 FDA approved drugs, and 2) 139,735 drug-like compounds to identify candidates for interacting with DNA binding domain of human RAD52. Thirty six lead candidate compounds were identified that were predicted to interfere with RAD52 –DNA binding. Further biological testing confirmed that 9 of 36 candidate compounds were able to inhibit the binding of RAD52 to single-stranded DNA *in vitro*. Based on molecular binding combined with functional assays, we propose a model in which the active compounds bind to a critical “hotspot” in RAD52 DNA binding domain 1. In addition, one of the 9 active compounds, adenosine 5’-monophosphate (A5MP), and also its mimic 5-aminoimidazole-4-carboxamide ribonucleotide (AICAR) 5’ phosphate (ZMP) inhibited RAD52 activity *in vivo* and exerted synthetic lethality against BRCA1 and BRCA2–mutated carcinomas. These data suggest that active, inhibitory RAD52 binding compounds could be further refined for efficacy and safety to develop drugs inducing synthetic lethality in tumors displaying deficiencies in BRCA1/2-mediated homologous recombination.

## Introduction

Since DNA repair mechanisms play a critical role in the induction, malignant progression and treatment of cancer [1], pharmacological targeting of tumor-specific DNA repair pathways may amplify endogenous and drug-induced DNA damage and trigger apoptosis in cancer cells. DNA double strand breaks (DSBs), the most lethal DNA lesions, are usually repaired by two major DSB repair pathways: homologous recombination repair (HRR) and non-homologous end-joining (NHEJ). While NHEJ plays a major role in non-proliferating cells, HRR works predominantly on broken replication forks and usually depends on BRCA1-PALB2-BRCA2-RAD51 paralogs (BRCA)–RAD51 pathway [2].

Enhanced self-renewal of cancer stem cells and high proliferation rate of cancer progenitor cells indicate that they use RAD51-dependent HR to repair spontaneous and therapy-induced DSBs [2]. RAD51 small molecule inhibitors have been recently developed [3], but their clinical application could be risky because of potential toxicity for normal cells [4] and/or facilitation of secondary tumors due to promotion of DSB repair by less faithful NHEJ or single-strand annealing (SSA) [5].

PARP1 inhibitors have been used to induce synthetic lethality in tumors harboring *BRCA1/2* mutations presumably by elevation of the number of DSBs in HR-impaired cancer cells [6]. Unfortunately, currently available PARP1 inhibitors do not discriminate between various PARPs [7]. Moreover, PARP1 inhibitors may generate serious side-effects because in addition to DNA repair, PARPs are involved in regulation of other functions, e.g., chromatin modification and transcriptional regulation [8].

However, in cells exhibiting genetic, epigenetic, and/or functional deficiencies in BRCA protein network, alternative HR mechanism such as RAD52-RAD51 pathway may emerge to protect cells from lethal effect of DSBs. RAD52 appears to be parallel with BRCA1/2 thus its inactivation should be synthetic lethal for *BRCA1/2*-mutated malignant cell lines [9]. In concordance, inhibition of RAD52 DNA binding activity by small peptide aptamer exerted synthetic lethality in BRCA1 and BRCA2 mutated cancer cells [10] and shRNA-mediated downregulation of RAD52 is lethal in tumor cell lines carrying BRCA2 inactivating mutations [9]. Since *Rad52* deletion in mice results only in mild phenotype without major impact on HR, and a peptide aptamer targeting RAD52 did not exert any detectable side effects in mice, RAD52 appears to be a promising target to trigger synthetic lethality in BRCA-deficient tumor cells without affecting normal cells [10, 11].

Peptide aptamers serve as guides for small molecule inhibitor (SMI) drug discovery [12], therefore these results strongly suggest that SMI targeting RAD52 DNA binding activity may exert synthetic lethality in wide range of BRCA-deficient tumors. As a step toward clinical applications we used structure-based selection combined with virtual computer screening and followed by functional assays to identify compounds capable to abrogate RAD52-ssDNA binding, inhibit RAD52 activity *in vivo* and exert synthetic lethality in BRCA-deficient cells.

## Materials and Methods

### Molecular docking

The crystal structure of human RAD52 [PDB code 1KN0 [13]] was used the basis for molecular docking. The site for molecular docking was selected using DMS (UCSF, San Francisco, CA) to generate a molecular surface, and SPHGEN_CPP to define spheres that represent sites of potential ligand atoms. Grid-based scoring implemented in DOCK6.6 was used. This was accomplished using a scoring grid extending 5 Å in 3 dimensions from the selected spheres. DOCK6.6 was used to screen a library of 1,217 FDA approved drugs (http://zinc.docking.org/catalogs/fda) and a library of 139,735 NCI drug-like compounds (http://zinc.docking.org/catalogs/ncip). Each drug/compound was docked in 1,000 orientations and scored for hydrophobic (van der Waals score) and electrostatic (electrostatic score) interactions at the University of Florida High Performance Computing Center by parallel processing using 8 cpu. Compounds were ranked based on overall Energy Score (van der Waals score + electrostatic score). 30 compounds were selected for further testing from the 100 top scoring compounds in the FDA approved drug library and the NCI drug-like library based on several characteristics: 1) most unique structurally, 2) easiest to manipulate chemically for lead optimization, and 3) most likely to be real interactions, based on the known electrostatic forces screened against.

### Docking of Confirmed Hits

Docking was performed using the tools available from Openeye Scientific Software (Sante Fe, NM). Structures of the confirmed hits were built and minimized with VIDA using the molecular builder and output in SMILES format. Starting from the published crystal structure of human RAD52 apoprotein (PDB code 1KNO (7)), the receptor was prepared using the Make Receptor utility in the OEdocking toolkit. Two adjacent monomers of the RAD52 oligomer were used for this purpose because the primary DNA binding site consists of a single continuous channel along the top surface of the oligomer. No H-bond or Van der Waals constraints were specified. The input database of bioactive conformations was prepared using the Omega toolkit with the DeleteFixHydrogens option and the option to generate all combinations of unspecified stereocenters. Output was in 3D sdf format. The FRED application was then used to dock the input database of the 9 confirmed hits and specify the top 5 poses per conformation.

### Experimental compounds

The top 30 scoring compounds from molecular docking were obtained from the NCI Developmental Therapeutic Program (DTP). AICAR (5-amino-1-((2R,3R,4S,5R)-3,4-dihydroxy-5-(hydroxymethyl)-tetrahydrofuran-2-yl)-1H-imidazole-4-carboxamide; CAS 2627-69-2) and AICAR 5’-phosphate [(2R,4R,5R)-5-(5-amino-4-carbamoylimidazol-1-yl)-3,4-dihydroxyoxolan-2-yl]methyl dihydrogen phosphate; AICA-Riboside, 5’-Phosphate = ZMP, CAS 3031-94-5] were purchased from Selleckchem and Calbiochem, respectively. F79 aptamer (Ac-VINLANEMFGYNG-GGG-YARAAARQARA-C(O)NH_2_) was prepared as previously described using fluorenylmethoxycarbonyl chemistry and microwave-assisted solid phase peptide synthesis [10].

### RAD52-ssDNA binding activity

The RAD52-ssDNA binding assay was performed as described before with modifications [10]. Briefly, 30-mer ssDNA oligonucleotide was HPLC purified and 5’ end-labelled with IRDye800 (5IRD800-AAG TGA ACA TAA AGT AAA TAA GTA TAA CGA) (Integrated DNA Technologies). 20 nM of IRDye800-labelled DNA substrate was incubated with GST-hRAD52 in specific binding buffer [10 mM Tris (pH 7.5), 50 mM KCl, 2.5 mM DTT/0.25% Tween 20, 0.1 mg/ml BSA]. Compounds selected by virtual computer screen (1–5 μM), ZMP (1 μM), and F79 aptamer (25 μM) were added to the reaction mixtures (20 μl), which were then incubated at 37°C for 15 minutes to allow for the formation of the DNA-protein complex. Glycerol was added to the samples at a final concentration of 2% before analysis on an 8% native polyacrylamide gel. The reaction products were imaged using an Odyssey Infrared Imager (LI-COR Biosciences). The presence of RAD52 protein in the reactions was confirmed by Western blot with anti-RAD52 antibody (Santa Cruz Biotechnology).

### GFP-RAD52 foci formation

BRCA1-deficient BCR-ABL1-32Dcl3 murine hematopoietic cells expressing GFP-RAD52 [10, 14] were pretreated or not for 4 hours with AICAR. Then, 3μg/mL cisplatin was added for 16 hours and cytospins were prepared using polylysine coated slides (Thermo Scientific). DNA was counterstained with DAPI. Foci were visualized with an inverted Olympus IX70 fluorescence microscope equipped with a Cooke Sensicam QE camera (The Cooke Co., Auburn Hills, MI, USA). Staining and images from 15 cells/group containing >5 foci/cell were processed using SlideBook 3.0 (Intelligent Imaging Innovation) as described before [10].

### RAD52-dependent SSA assay

U2OS cells with SSA reporter cassette (SA-GFP) were obtained from J. Stark [15]. Cells (2 × 10^5^) were plated in triplicate in 12 well plates, and treated with 0, 10, and 20 μM AICAR. Cells were transfected 24 hours later with 0.8 μg pCβA-Sce expression plasmid encoding I-SceI endonuclease and 0.8 μg pDsRed1-Mito (Clontech, Paolo Alto, CA) using Lipofectamine 2000 (Invitrogen). Second dose of AICAR was added immediately after transfection. SSA activity was measured 72 hours post-transfection and was determined as percentage of GFP+/DsRed1+ cells in DsRed1+ cells.

### Testing the compounds in BRCA–deficient cells

Breast cancer cell line, HCC1937, (5382insC germ-line mutation generating the truncated protein and no wild-type allele, BRCA1-null) and cells with restored BRCA1 expression (BRCA1-reconstituted) were obtained from Dr R. Scully (Beth Israel Deaconess Medical Center and Harvard Medical School, Boston, MA, USA) [16]. Human pancreatic adenocarcinoma Capan-1 cells carrying *BRCA2* has a 6174delT mutation, which causes a premature C-terminal truncation that removes the domains for DNA repair and the nuclear localization signals, and their BRCA2 wild-type reconstituted counterparts were obtained from Dr S. Powell (Memorial Sloan-Kettering Cancer Center, New York, NY) [10]. BRCA1-deficient BCR-ABL1-32Dcl3 murine hematopoietic cells and their counterparts expressing ectopic BRCA1 were described before [10]. Cells were treated with the compounds on day 0, +2 and +4, and counted in Trypan blue on day +5.

## Results and Discussion

**Fig 1:**
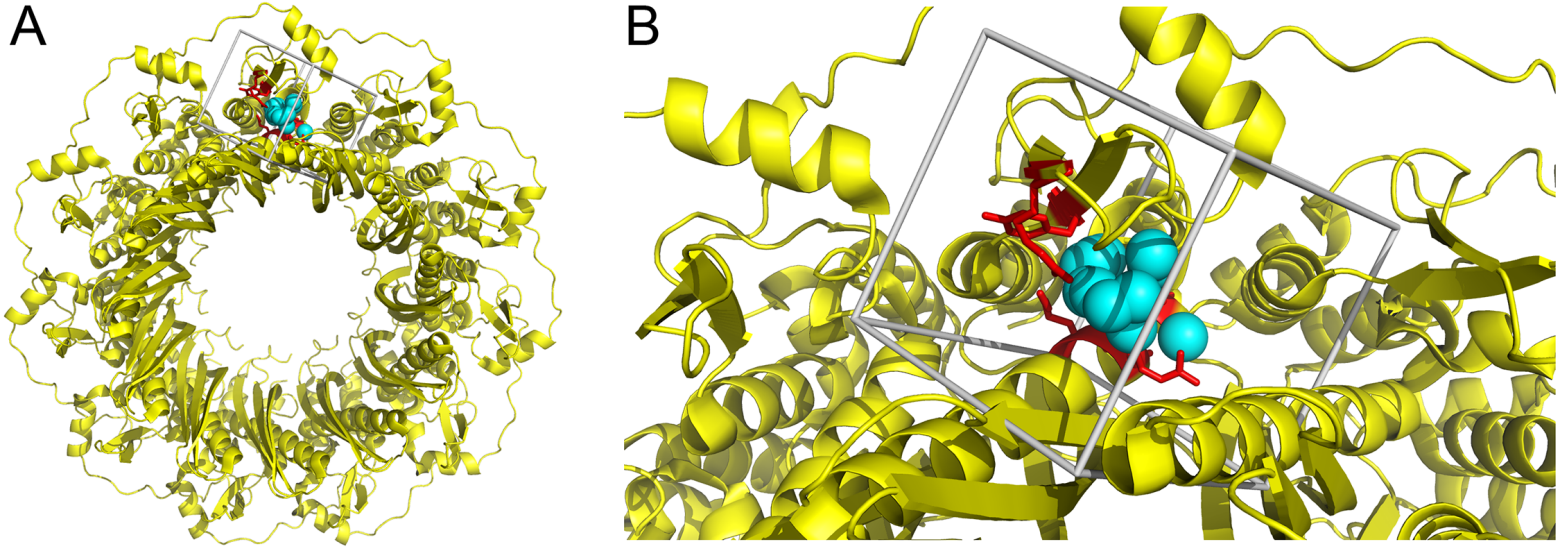
Structure-based strategy to select RAD52 inhibitors. (**A**) The crystal structure of RAD52 is shown in yellow as a ribbon diagram. The DNA binding residues identified in the crystal structure, pdb 1KN0, are shown in red (ARG55, TYR65, LYS152, ARG153, ARG156). The sites selected for molecular docking is shown in cyan as spheres. (**B**) DNA binding residues of RAD52 and the site selected for molecular docking surrounded by box depicting the boundaries of the scoring grid used for molecular docking.

RAD52 is a complex formed by the oligomerization of 7–11 individual monomers. The apo crystal structure of the 11-mer has been solved and a specific structural site containing R55, Y65, K152, R153, R156 is implicated in direct DNA binding [13]. We used the crystal structure of human RAD52 as the basis for selection of compounds that may interfere with DNA binding [13] by *in silico* molecular docking. R55, Y65, K152, R153, R156 were selected as a potentially druggable site for molecular docking using the DOCK6.6 program package [17, 18]. A molecular surface was drawn over the crystal structure of RAD52 and spheres representing potential ligand atoms were placed in concave structural pockets. Spheres within 6 Å of R55, Y65, K152, R153, R156 were used to describe the site for molecular docking. Each compound was docked into the selected site, shown in **Fig 1**, in 1,000 orientations and scored based on predicted H-bond and van der Waals contacts.

**Fig 2:**
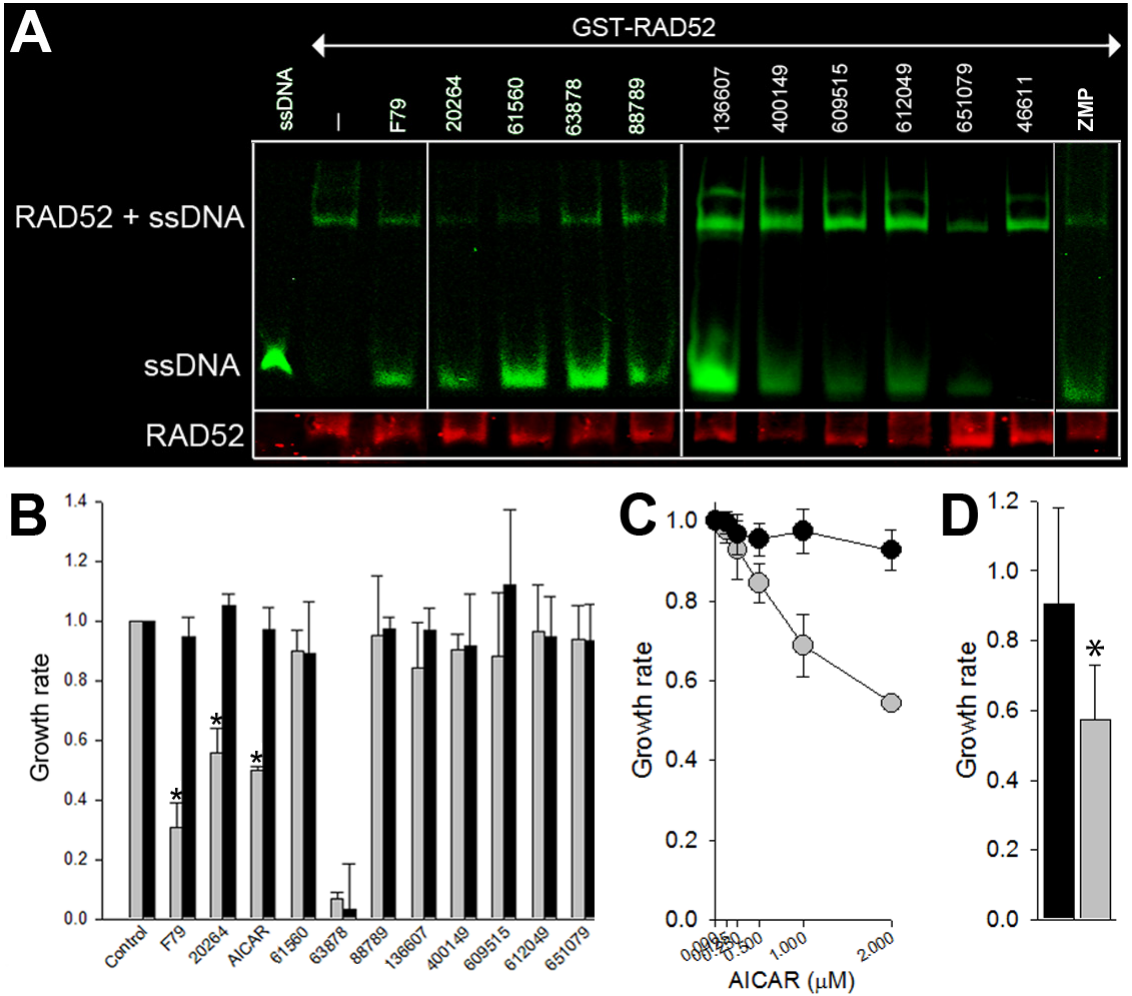
Screening for inhibitors of RAD52-ssDNA exerting synthetic lethality in BRCA-deficient cells. (**A**) The indicated compounds, F79 aptamer (F79), or DMSO (-), were added to the mixture of GST-RAD52 and IRDye800-ssDNA (ssDNA = IRDye800-ssDNA only). RAD52-ssDNA complex, free ssDNA, and RAD52 protein were detected by fluorescence and Western analysis, respectively. Compound 46611 was used as negative control. Results represent at least 3 experiments. (**B**) Growth of BRCA1-null HCC1937 cells (grey bars) and their BRCA1-reconstitued counterparts (black bars) in the presence of diluent, 5 μM F79 and indicated compounds (10 μM of 63878 and 20 μM of the others). Number of cells ± SD normalized to untreated controls from 3 experiments in triplicates, *p<0.01. (**C**) Growth of BRCA1-null HCC1937 cells (grey dots) and their BRCA1-reconstitued counterparts (black dots) in the presence of indicated concentrations of AICAR. Number of cells ± SD normalized to untreated controls from 2 experiments in triplicates. (**D**) Growth of BRCA2 mutated human pancreatic adenocarcinoma Capan-1 cells (grey bar) and their BRCA2 wild-type reconstituted counterparts (black bar) in the presence of 20 μM AICAR. Number of cells ± SD normalized to untreated controls from 2 experiments in triplicates, *p<0.03.

The 100 highest scoring compounds from 139,735 compounds in the NCI DTP plated set (based on the overall energy score) and 1,217 FDA approved drugs were filtered to define a set representing: 1) the most unique structurally, 2) easiest to manipulate chemically for lead optimization, and 3) most likely to be accurate posed interactions. 30 compounds were selected using these criteria from the top 100 scoring compounds from each library (i.e., 30 selected from 200 top scoring compounds) for functional testing. Nine of 30 pre-selected compounds interfered with RAD52-ssDNA binding *in vitro* (**Fig 2A**).Therefore these compounds are confirmed hits for biochemical inhibitor activity and can serve as templates for optimization (**Table 1**).

**Table 1:** Small molecule inhibitors of RAD52 –ssDNA binding *in vitro.*

Compound name	Chemical formula	Molecular weight	NCI number	Docking score
A5MP	C_10_H_14_N_5_O_7_P	347.2237	NSC20264	-39.98
N-(dichloroacetyl)aspartic acid (ACD/Name 4.0)	C_6_H_7_Cl_2_NO_5_	244.031	NSC136607	-39.91
2-(4-benzyl-2,5-dioxo-1-imidazolidinyl)-3-phenylpropanoic acid (ACD/Name 4.0)	C_19_H_18_N_2_O_4_	338.3622	NSC609515	-38.66
((1,3,9-trimethyl-2,6-dioxo-2,3,6,9-tetrahydro-1H-purin-8-yl)thio)acetic acid (ACD/Name 4.0)	C_10_H_12_N_4_O_4_S	284.2892	NSC400149	-36.98
6-(1-aziridinyl)-7-methyl-5,8-dioxo-2,3,5,8-tetrahydro-1H-pyrrolo[1,2-a]benzimidazol-3-yl propionate (ACD/Name 4.0)	C_16_H_17_N_3_O_4_	315.328	NSC651079	-36.96
9-(5-(hydroxymethyl)tetrahydro-2-furanyl)-9H-purin-6-ol (ACD/Name 4.0)	C_10_H_12_N_4_O_3_	236.2298	NSC612049	-28.22
u-19920a	C_9_H_13_N_3_O_5_	243.2188	NSC63878	-27.89
6-chloro-3-(dichloromethyl)-3,4-dihydro-2H-1,2,4-benzothiadiazine-7-sulfonamide 1,1-dioxide (ACD/Name 4.0)	C_8_H_8_Cl_3_N_3_O_4_S_2_	380.6479	NSC61560	-26.36
6-(((1-aminocyclohexyl)carbonyl)amino)-3,3-dimethyl-7-oxo-4-thia-1-azabicyclo[3.2.0]heptane-2-carboxylic acid (ACD/Name 4.0)	C_15_H_23_N_3_O_4_S	341.4244	NSC88789	-23.70

NSC20264, NSC63878, NSC61560, and NSC88789 are FDA approved drugs.

Comparison of active (confirmed hits) versus non-active compounds showed structural differences. For example, out of those 30 tested, 33% of the active compounds had characteristics of nucleotide analogs (3 out of 9), whereas only 14% of the inactive compounds had characteristics of nucleotide analogs (3 out of 21). These data suggest that molecular docking predicted binding modes of the nucleotide analogs that may provide the basis for rational modification and serve as templates for optimization.

One of 9 confirmed hits, National Cancer Institute NSC 20264 (adenosine 5’-monophosphate, A5MP), inhibited the growth of BRCA1-mutated HCC1937 breast carcinoma cells, but not the cells with restored BRCA1 expression (**Fig 2B**). Extracellular A5MP is de-phosphorylated to adenosine, which is then transported in to the cells and re-phosphorylated to A5MP (**Figure A in S1 File**).

5-Aminoimidazole-4-carboxamide ribonucleotide (AICAR) 5’ phosphate (ZMP), a compound known to mimic A5MP, also disrupted RAD52-ssDNA interaction (**Fig 2A**), but it did not disturb RAD51-ssDNA complex formation and did not cause DNA intercalation (**Figure B in S1 File**). ZMP is not cell-permeable, but cell-permeable AICAR undergoes spontaneous phosphorylation in the cytoplasm causing accumulation of ZMP in cells (**Figure A in S1 File**). Similar to A5MP, AICAR preferentially eliminated BRCA1-mutated HCC1937 breast carcinoma cells and BRCA2-mutated Capan-1 pancreatic adenocarcinoma cells when compared to BRCA1 or BRCA2-reconstituted counterparts (**Fig 2B–2D**).

It has been suggested that AICAR can affect cell proliferation/survival by activation of AMP-activated protein kinase (AMPK) [19]. However AICAR-induced anti-proliferative effect was abrogated by ectopic expression of BRCA1 in BRCA1-mutated HCC1937 cells (**Fig 2B and 2C**) and in BRCA1-deficient BCR-ABL1 –positive leukemia cells (**Figure C in S1 File**), implicating synthetic lethal effect. Moreover, while AICAR reduced the growth of BRCA1-deficient BCR-ABL1 –positive leukemia cells, it was ineffective in cells lacking RAD52 (**Figure C in S1 File**) [10].

**Fig 3:**
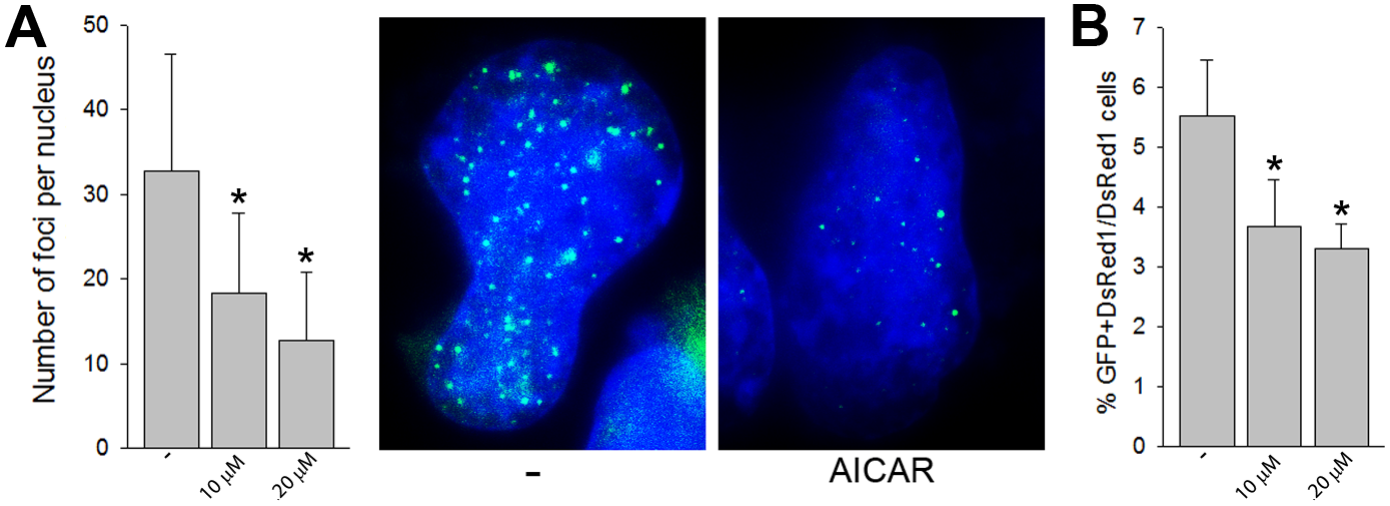
AICAR inhibits RAD52 activity in cells. (**A**) Number of GFP-RAD52 foci/nucleus (10–30 nuclei/group from 2 experiments) in cisplatin-treated BCR-ABL1 –positive BRCA1-deficient cells pre-incubated, or not, with 10 and 20 μM AICAR; *p<0.001 in comparison to control (-). Representative nuclei with GFP-RAD52 foci from cisplatin-treated cells pre-incubated, or not, with 20 μM AICAR. (**B**) Percentage of double positive GFP+DsRed1+ cells (RAD52-mediated SSA restored expression of GFP) in DsRed1 population representing SSA activity in U2OS cells treated, or not, with 10 and 20 μM AICAR in 5 independent experiments, *p≤0.004 in comparison to control (-).

To confirm that AICAR targets intracellular RAD52 we demonstrated that the compound reduced GFP-RAD52 nuclear foci induced by cisplatin in BRCA1-deficient BCR-ABL1 –positive leukemia cells (**Fig 3A**) [14]. In addition, we examined the effects of AICAR on SSA which is one of the main activities of RAD52 [13, 20]. To measure SSA, a previously characterized GFP reporter system was used which detects activation of GFP expression as a result of SSA repair [15]. AICAR applied in concentrations which reduced RAD52 foci formation, inhibited SSA activity (**Fig 3B**). Altogether, these data support the speculation that A5MP and its analogue AICAR/ZMP may target RAD52 to exert synthetic lethality in BRCA1-deficient tumor cells [9].

**Fig 4:**
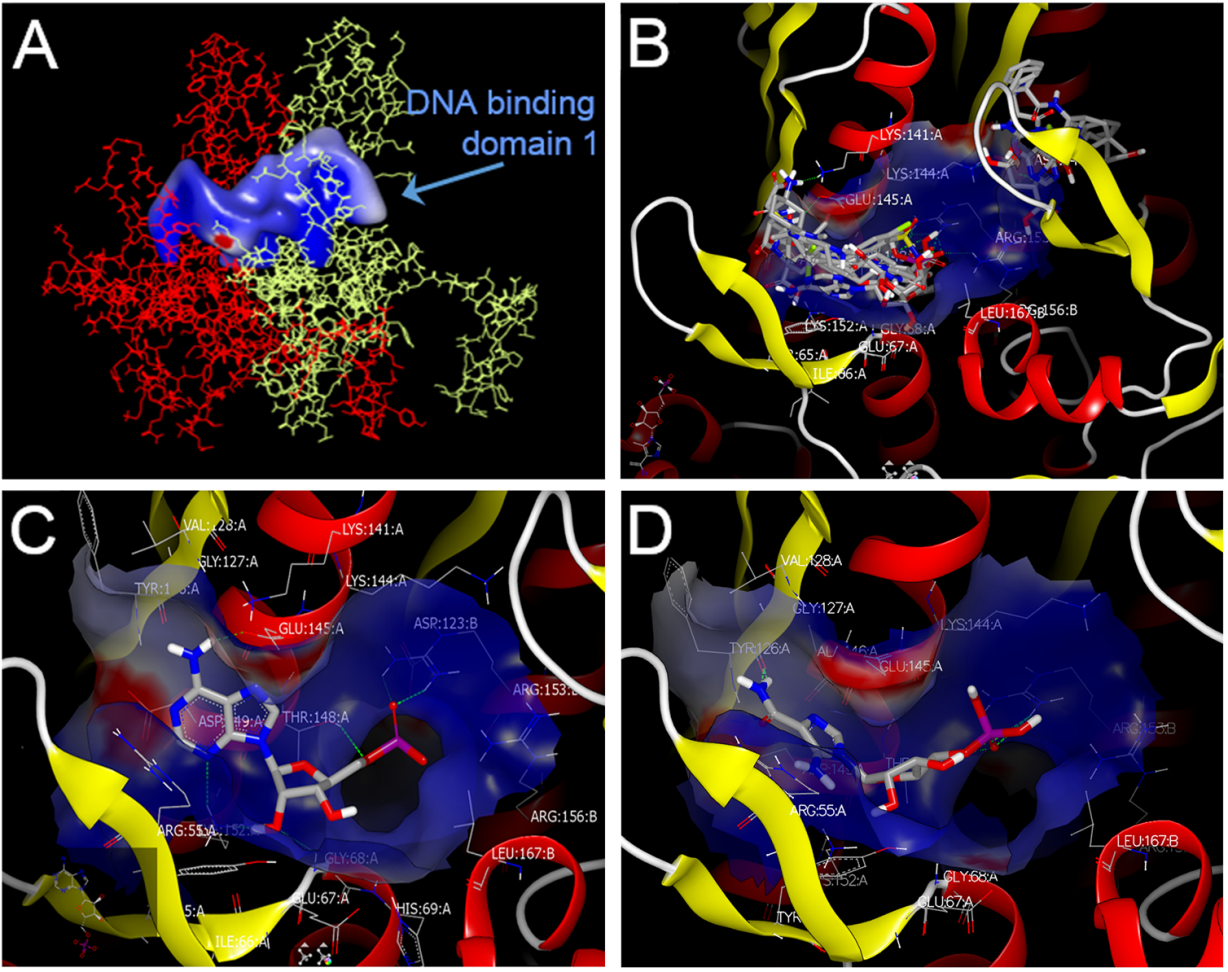
Modeling of the interaction between RAD52 DNA binding domain 1 and candidate inhibitory compounds. (**A**) The DNA binding site 1 of RAD52 as identified by OEDocking (Openeye). The continuous channel of DNA binding site 1 as formed by adjacent monomers in red and yellow. (**B**) Proposed “hotspots” for binding of the 9 “confirmed hits” in DNA binding domain 1. These “hits” were docked to the DNA binding domain 1 of a RAD52 dimer using OEDocking (Openeye) to establish a docking model. (**C,D**) The predicted binding mode of the RAD52 primary DNA binding site 1 using the docked ligands: A5MP (**C**) and AICAR 5’-phosphate (ZMP) (**D**).

Docking (OEdocking from Openeye) was used to pinpoint the potential binding sites of the confirmed hits and ZMP. Two adjacent monomers of the RAD52 oligomer were used for this purpose because the primary binding site consists of a single continuous channel along the top surface of the oligomer. DNA binding site 1 sits in the continuous channel along the top of the two monomers (**Fig 4A**) and DNA binding site 2 is located towards the edge (**Figure D in S1 File**). These two DNA binding sites have been previously described [20, 21]. According to our docking model all 9 confirmed hits bind to the hotspot in RAD52 DNA binding domain 1 (**Fig 4B**).

Analysis of the docking results with A5MP (**Fig 4C**) and ZMP (**Fig 4D**) suggests that the binding site for both molecules lies within DNA binding domain 1 (**Fig 4A**) at the junction between two monomers of RAD52 (subsequently referred to as subunits A and B). The two molecules bind in a similar conformation, with the amide group of ZMP forming similar interactions with the protein as those seen with the aminopyrimidine moiety of A5MP. The phosphate groups of both compounds assume a similar orientation between arginine 153 (ARG155) and ARG156 on the B subunit of RAD52, making one hydrogen bond interaction with ARG155B and another hydrogen bond with threonine 148 on the A subunit (THR148A). More detailed analysis of the docking model of ZMP (**Figure E in S1 File**) reveals that the furan oxygen of the ribose moiety does not appear to make any interactions with the protein, although it is fairly close to the side-chain NH_2_ of lysine 152 A (LYS152A) (approximately 3.8 Å). The ribose OH groups are pointed toward solvent. The amide NH_2_ moiety of ZMP appears to make a hydrogen bond with the backbone carbonyl group of THR126A. The carbonyl oxygen of that amide group points toward the guanidine group of arginine 55A (ARG55A) but would require movement by the ARG55A side chain to form hydrogen bonds that were not seen in these docking studies.

A number of sites on ZMP offer the possibility of structural modification to enhance its intracellular anti-RAD52 activity. For example, a phosphate bioisostere approach might represent a viable strategy for designing a permeable ZMP analog that is potent, selective for the DNA binding of RAD52 and bioavailable.

In the absence of active transport, small molecule drugs must rely on diffusion to enter a cell to reach an intracellular biological target like RAD52. AICAR, a permeable molecule, diffuses into cells where it is phosphorylated to give the active species ZMP, a non-permeable molecule [22]. Many properties of a molecule contribute to its cellular permeability; however, lipophilicity is one important determinant. As a general rule, compounds displaying good passive diffusion across biological membranes tend to have logP values > 0.8 [23]. Of the four most potent hits (20604 (A5MP), 63878, 61560 and 88789, **Fig 2A**), only 61560 (trichlomethiazide, a diuretic agent) possesses a positive logP value (**Table A in S1 File**). Given the acceptable *in vitro* physicochemical properties of 61560 (logP = 0.95, topological polar surface area = 118.4), the fact that the chemical scaffold has already been proven safe in humans and the limited drug discovery efforts published on the scaffold (changes in the aryl sulfonamide and exchange of fluorine for chlorine in the dimethylchloro group), 61560 could represent a reasonable starting point for medicinal chemistry aimed at further improving physicochemical properties and eliminating diuretic activity. One other hit, 609515, also possesses reasonable physicochemical properties (logP = 2.31, topological polar surface area = 87.7) and even showed a trend toward selectively inhibiting BRCA1 mutated cells versus cells with restored BRCA1 activity (**Fig 2B**). In addition to inhibiting ssDNA binding to RAD52, compounds possessing the benzylhydantoin chemical scaffold of 609515 are known to be inhibitors of Bcl-2 [24] and dihydroorotate dehydrogenase [25], two other potential anti-cancer mechanisms. This hit might represent a starting point for a multi-mechanism antineoplastic hit to lead project.

An additional docking calculation was performed using several hundred bromine-containing small fragments (**Figure F in S1 File**). The purpose of this step was to flood the RAD52 DNA binding site 1 with a diverse set of functional groups in order to find “hotspot” residues, and to use these hotspots as guides for future drug design. An overlap of these fragments indicates that there may be multiple binding modes in the channel that may be exploited using library design and bioisosteric replacement tools. This approach has been applied successfully to identify hits using x-ray crystallography and nuclear magnetic resonance [26–28]. A number of possible interaction sites were identified based on the docking of the bromine-containing fragments. In particular, ARG55 and LYS141 are potential target residues for additional H-bond interactions while the fragments cluster into two distinct areas, indicating multiple potential binding modes.

In summary, we applied virtual computer screens of the Food and Drug Administration (FDA) approved drug library and National Cancer Institute (NCI) drug-like library combined with RAD52 functional assay and BRCA-dependent anti-tumor effect to indentify compound(s) capable of disrupting ssDNA binding by RAD52 and/or exerting anti-tumor activity against BRCA-mutated carcinoma cells. Some of these hits may be further developed as anti-RAD52 drugs that induce synthetic lethality to treat patients with BRCA-deficient tumors (**Figure G in S1 File**). Potential application of RAD52 small molecule inhibitors as future drugs is supported by recent report that 6-hydroxy-DL-dopa disrupted RAD52 ring formation causing synthetic lethality in BRCA-deficient tumor cells [29].

## Supporting Information

S1 File Figures A-G, Table A. (PDF)
