# Supplementary material for: Identification of a Small Molecule Inhibitor of RAD52 by Structure-Based Selection
Source: PLoS One. 2016 Jan 19;11(1):e0147230. doi: 10.1371/journal.pone.0147230 (PMC4718542; doi:10.1371/journal.pone.0147230)
Supplement: S1 File — (PDF) [file pone.0147230.s001.pdf]

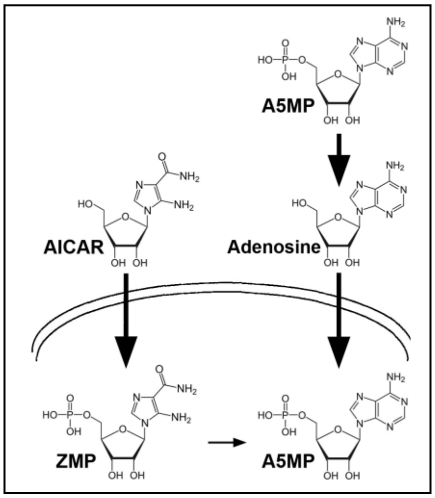

Supplemental Figure A. AICAR, ZMP and A5MP. AICAR is transported across the plasma membrane and rapidly phosphorylated to ZMP in the cytoplasm following by slow metabolism to A5MP [1]. Extracellular A5MP is split to adenosine, which is transported in to the cells and re-phosphorylated to A5MP [2, 3].

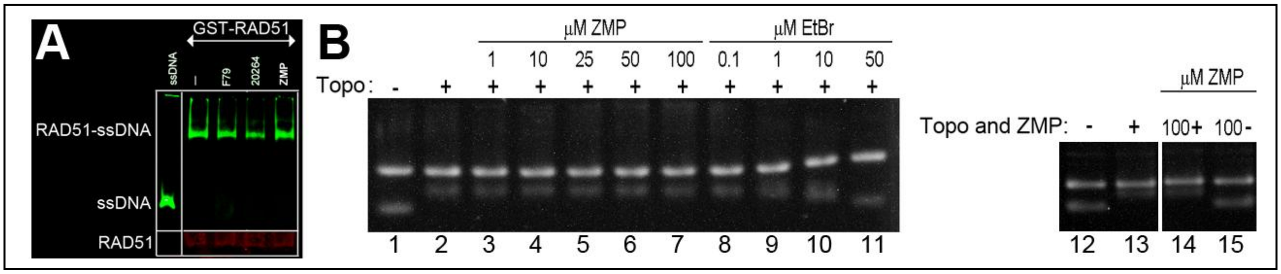

Supplemental Figure B. RAD52 inhibitors do not affect RAD51 – ssDNA binding and did not intercalate DNA. (**A**) F79 aptamer (F79), A5MP (20264), ZMP, or DMSO (-) were added to the mixture of GST-RAD51 and IRDye800-ssDNA (ssDNA = IRDye800-ssDNA only). The DNA binding reaction was performed as described for GST-RAD52 with the following modification: specific binding buffer was supplemented with 1mM ATP and 2mM $MgCl_2$ [4]. RAD51-ssDNA complex, free ssDNA, and RAD51 protein were detected by fluorescence and Western analysis, respectively (2 experiments). (**B**) DNA intercalation activity was measured using DNA unwinding kit (TopoGEN, Inc., Buena Vista, CO, USA). Briefly, pHOT1 supercoiled plasmid (lanes 1, 12) was relaxed with topoisomerase I (Topo; lanes 2, 13) followed by addition of ZMP (lanes 3-7) or ethidium bromide (EtBr, positive control; lanes 8-11) for 30 min. Reactions were terminated by 1% SDS, digested by proteinase K, and analyzed in 1% agarose gel. ZMP did not intercalate DNA in contrast to EtBr. In addition, 100 μM ZMP was added simultaneously with Topo to demonstrate that it did not inhibit the enzyme (lane 14). Moreover 100 μM ZMP, when added to the plasmid, did not nick the substrate (lane 15).

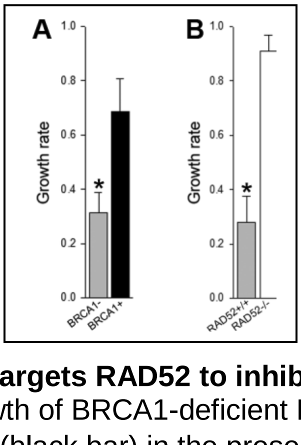

Supplemental Figure C. AICAR targets RAD52 to inhibit growth of BRCA1-deficient BCR-ABL1 – positive leukemia cells. (**A**) Growth of BRCA1-deficient BCR-ABL1 –positive 32Dcl3 cells (grey bar) and their BRCA1-reconstitued counterparts (black bar) in the presence of AICAR (10 μM). Results represent number of cells ± SD normalized to untreated controls in 3 independent experiments, *p<0.01. (**B**) AICAR abrogated colony forming activity of BRCA1-deficient BCR-ABL1 *Rad52+/+* leukemia cells (grey bar), but not their *Rad52-/-* counterparts (white bar). Results represent number of cells ± SD normalized to untreated controls from 3 experiments, *p<0.001.

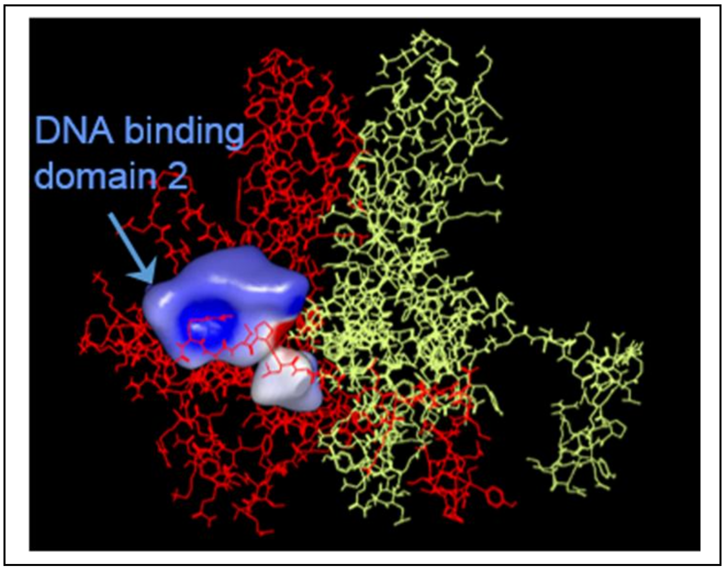

Supplemental Figure D. DNA binding site 2, which while not continuous between the two monomers is formed from residues from both monomers.

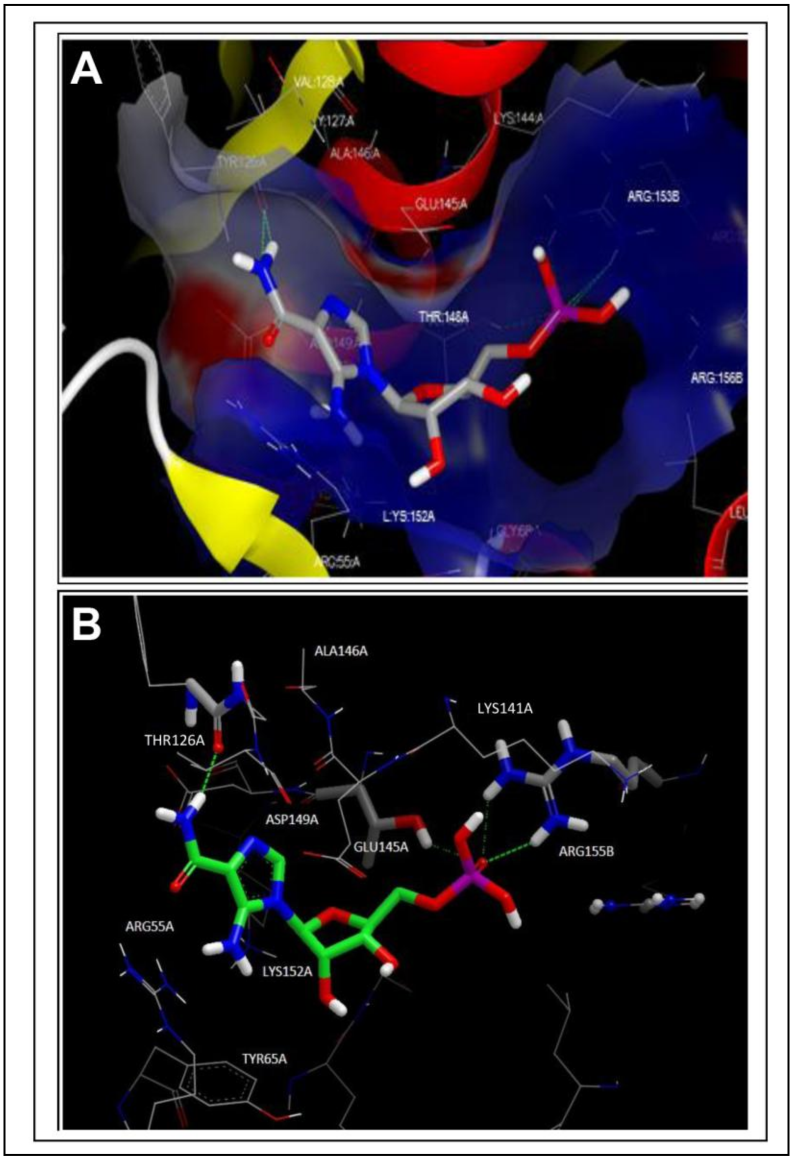

Supplemental Figure E. ZMP docked to human RAD52 DNA binding domain (PDB 1KNO). (A) Docking was performed using the tools available from Openeye Scientific Software (Sante Fe, NM). The structure of ZMP was built and minimized with VIDA. Starting from the published crystal structure of human RAD52 apoprotein [PDB code 1KNO (7)], the receptor was prepared using the Make Receptor utility in the OEdocking toolkit. Two adjacent monomers of the RAD52 oligomer were used for this purpose because the primary DNA binding site consists of a single continuous channel along the top surface of the oligomer. No H-bond or Van der Waals constraints were specified. The input database of bioactive conformations was prepared using the Omega toolkit with the DeleteFixHydrogens option and the option to generate all combinations of unspecified stereocenters [5]. Output was in 3D sdf format. The Fred application was then used to dock ZMP [6]. **(B)** ZMP docked into its proposed binding site on RAD52. Hydrogen bonding interactions with THR126A, THR148A and ARG155B are shown as dotted lines.

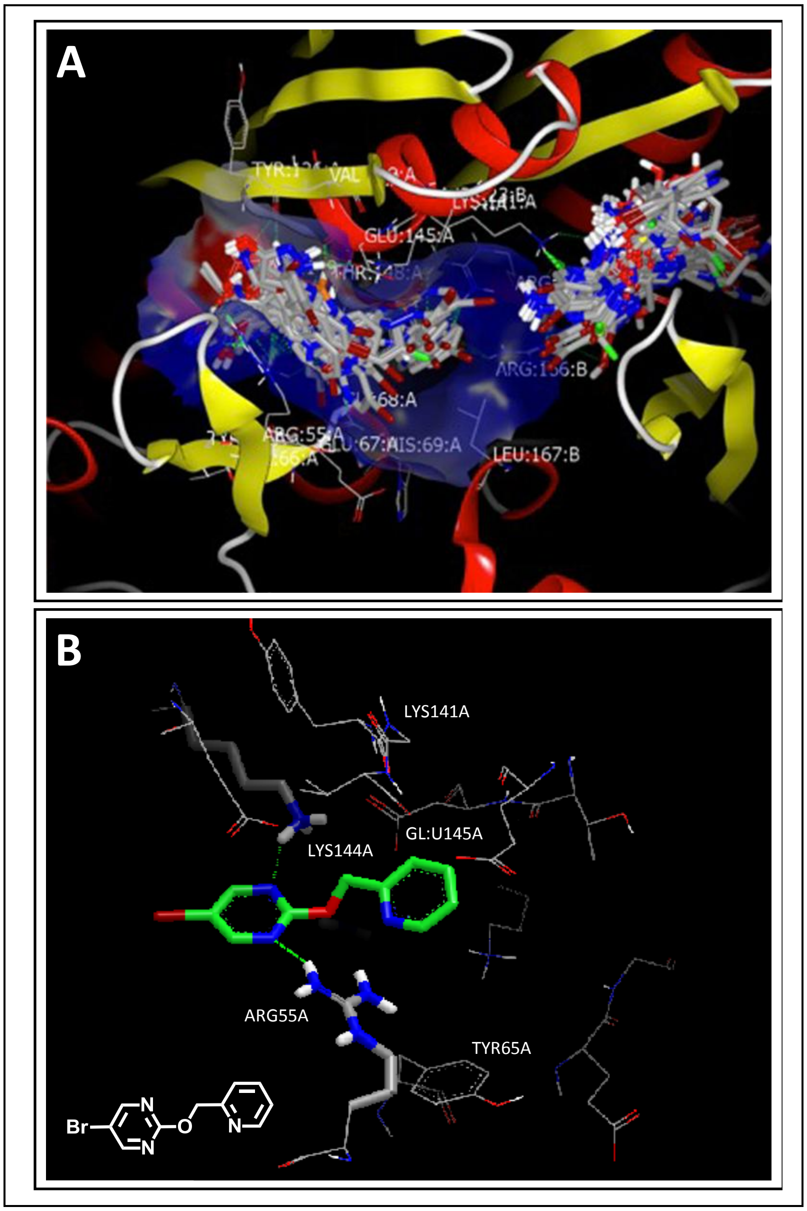

Supplemental Figure F. Small bromine fragments docked in the DNA binding site 1 of RAD52. (**A**) Two hundred small, non-reactive bromine-containing fragments were selected from commercially vendor libraries (Maybridge, Otava, Life Chemicals) based on structural diversity, prepared and docked with the RAD52 receptor model described above (Supplemental Figure 4). All bromine fragments met the Congreve "rule of three" for fragment-based screening [7]. (**B**) Example of docked small bromine fragment (5-bromo-2-(pyridine-2-yl)methoxypyrimidine) indicating that ARG55A and LYS144A are exposed in the binding pocked and available for interaction with RAD52 inhibitor ligands.

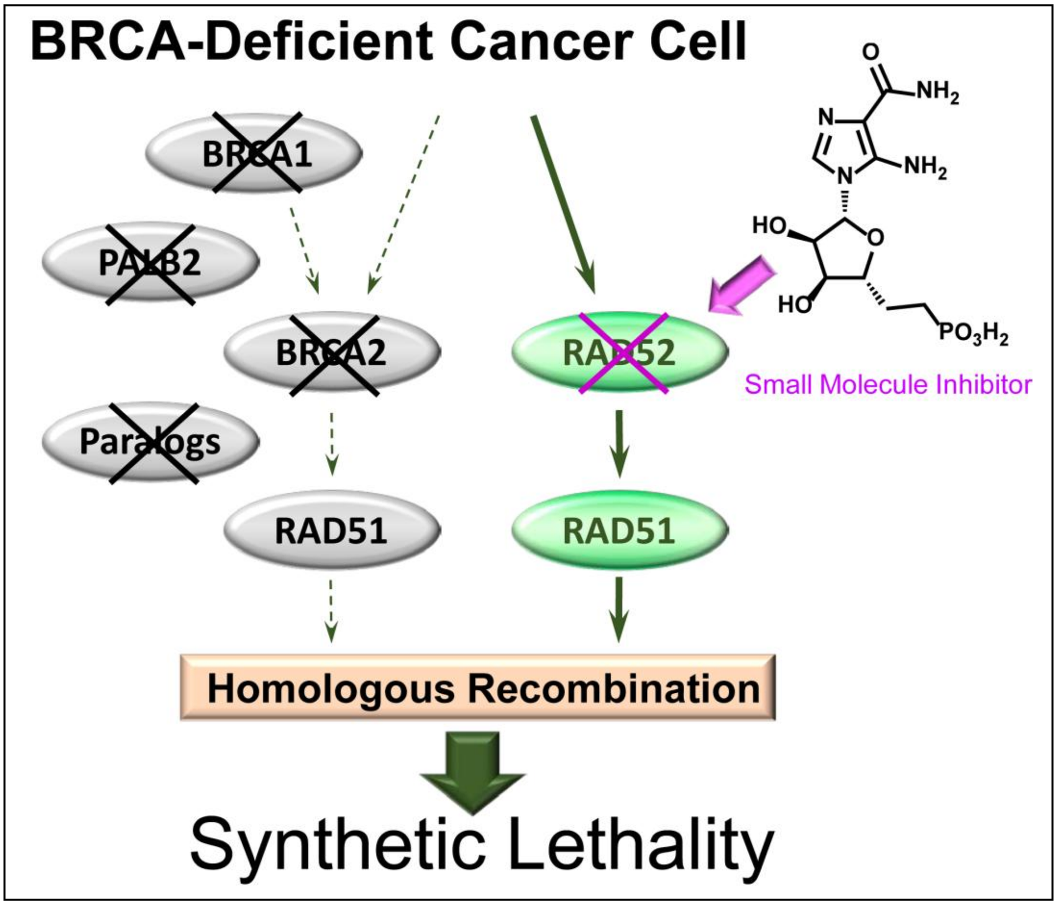

Supplemental Figure G. Small molecule inhibitor of RAD52 induces synthetic lethality in BRCA-deficient cells.

Supplemental Table A. logP and topological polar surface area values for RAD52 inhibitor hits.

Structure	NSC Number	logP	TPSA*	Most Potent Inhibitors†
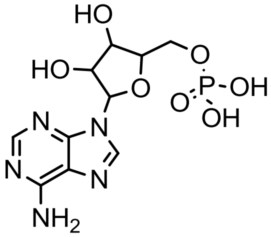	20604 (A5MP)	-3.50	182.8	√
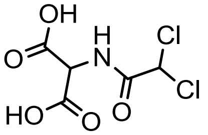	136607	-0.52	103.7	
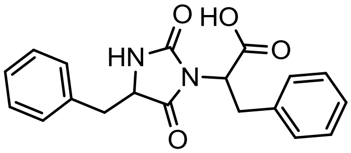	609515	2.31	87.7	
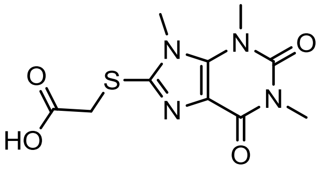	400149	-0.27	93.5	
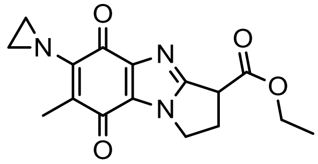	651079	-0.8	79.1	
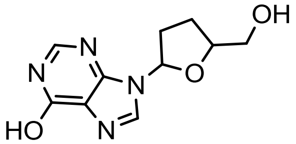	612049	0.08	90.0	
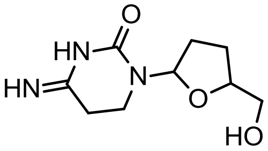	63878	-0.89	85.7	√
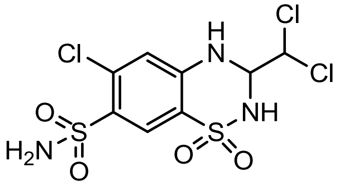	61560	0.95	118.4	√
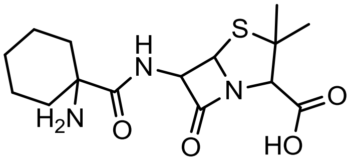	88789	-0.31	112.7	√

* TPSA = Topological Polar Surface Area; †The four most potent inhibitors of ssDNA binding to RAD52.

SUPPLEMENTAL REFERENCES

1. Corton JM, Gillespie JG, Hawley SA, Hardie DG. 5-aminoimidazole-4-carboxamide ribonucleoside. A specific method for activating AMP-activated protein kinase in intact cells? Eur J Biochem. 1995;229(2):558-65. PubMed PMID: 7744080.
2. Weisman GA, Lustig KD, Lane E, Huang NN, Belzer I, Friedberg I. Growth inhibition of transformed mouse fibroblasts by adenine nucleotides occurs via generation of extracellular adenosine. J Biol Chem. 1988;263(25):12367-72. PubMed PMID: 2842330.
3. Rapaport E. Compartmentalized ATP pools produced from adenosine are nuclear pools. J Cell Physiol. 1980;105(2):267-74. doi: 10.1002/jcp.1041050210 [doi]. PubMed PMID: 6450772.
4. Tombline G, Heinen CD, Shim KS, Fishel R. Biochemical characterization of the human RAD51 protein. III. Modulation of DNA binding by adenosine nucleotides. J Biol Chem. 2002;277(17):14434-42. doi: 10.1074/jbc.M109917200 [doi]
M109917200 [pii]. PubMed PMID: 11839741.
5. Bostrom J, Greenwood JR, Gottfries J. Assessing the performance of OMEGA with respect to retrieving bioactive conformations. J Mol Graph Model. 2003;21(5):449-62. doi: S1093-3263(02)00204-8 [pii]. PubMed PMID: 12543140.
6. McGann M. FRED and HYBRID docking performance on standardized datasets. J Comput Aided Mol Des. 2012;26(8):897-906. doi: 10.1007/s10822-012-9584-8 [doi]. PubMed PMID: 22669221.
7. Congreve M, Carr R, Murray C, Jhoti H. A 'rule of three' for fragment-based lead discovery? Drug Discov Today. 2003;8(19):876-7. doi: S1359644603028319 [pii]. PubMed PMID: 14554012.
